# A comparison between measured and calculated central venous oxygen saturation in critically ill patients

**DOI:** 10.1371/journal.pone.0206868

**Published:** 2018-11-08

**Authors:** Bruno De Oliveira, Malligere Prasanna, Malcolm Lemyze, Laurent Tronchon, Didier Thevenin, Jihad Mallat

**Affiliations:** 1 Department of Critical Care Medicine, Critical Care Institute, Cleveland Clinic Abu Dhabi, Abu Dhabi, UAE; 2 Department of Anesthesiology and Critical Care Medicine, Centre Hospitalier du Dr. Schaffner de Lens, Lens, France; University of Notre Dame Australia, AUSTRALIA

## Abstract

**Background:**

Central venous oxygen saturation (ScvO_2_) is often used to help to guide resuscitation of critically ill patients. The standard gold technique for ScvO_2_ measurement is the co-oximetry (Co-oximetry_ScvO_2_), which is usually incorporated in most recent blood gas analyzers. However, in some hospitals, those machines are not available and only calculated ScvO_2_ (Calc_ScvO_2_) is provided. Therefore, we aimed to investigate the agreement between Co-oximetry_ScvO_2_ and Calc_ScvO_2_ in a general population of critically ill patients and septic shock patients.

**Methods:**

A total of 100 patients with a central venous catheter were included in the study. One hundred central venous blood samples were collected and analyzed using the same point-of-care blood gas analyzer, which provides both the calculated and measured ScvO_2_ values. Bland and Altman plot, intra-class correlation coefficient (ICC), and Cohen’s Kappa coefficient were used to assess the agreement between Co-oximetry_ScvO_2_ and Calc_ScvO_2_. Multiple linear regression analysis was performed to investigate the independent explanatory variables of the difference between Co-oximetry_ScvO_2_ and Calc_ScvO_2_.

**Results:**

In all population, Bland and Altman’s analysis showed poor agreement (+4.5 [-7.1, +16.1]%) between the two techniques. The ICC was 0.754 [(95% CI: 0.393–0.880), P< 0.001], and the Cohen’s Kappa coefficient, after categorizing the two variables into two groups using a cutoff value of 70%, was 0.470 (P <0.001). In septic shock patients (49%), Bland and Altman’s analysis also showed poor agreement (+5.6 [–6.7 to 17.8]%). The ICC was 0.720 [95% CI: 0.222–0.881], and the Cohen’s Kappa coefficient was 0.501 (P <0.001). Four independent variables (PcvO_2_, Co-oximetry_ScvO_2_, venous pH, and Hb) were found to be associated with the difference between the measured and calculated ScvO_2_ (adjusted R^2^ = 0.8, P<0.001), with PcvO_2_ being the main independent explanatory variable because of its highest absolute standardized coefficient. The area under the receiver operator characteristic curves (AUC) of PcvO_2_ to predict Co-oximetry_ScvO_2_ ≥ 70% was 0.911 [95% CI: 0.837–0.959], in all patients, and 0.903 [95% CI: 0.784–0.969], in septic shock patients. The best cutoff value was ≥ 36 mmHg (sensitivity, 88%; specificity, 83%), in all patients, and ≥ 35 mmHg (sensitivity, 94%; specificity, 71%) in septic shock patients.

**Conclusions:**

The discrepancy between the measured and calculated ScvO_2_ is clinically not acceptable. We do not recommend the use of calculated ScvO_2_ to guide resuscitation in critically ill patients. In situations where the Co-oximetry technique is not available, relying on PcvO_2_ to predict the measured ScvO_2_ value above or below 70% could be an option.

## Introduction

Ensuring adequate oxygen delivery to organs and tissues is one of the primary objectives of organ support and goal-directed strategies in critical care. There are no readily available methods to monitor oxygen delivery to tissues directly in daily practice, and so physicians must instead rely on indirect measurements such as venous oxygen saturation.

Venous oxygen saturation is commonly used in the evaluation of patients in the intensive care unit (ICU) and may be of value in the management of septic patients [1] and post-cardiac surgery patients [2].

Mixed venous oxygen saturation (SvO_2_) obtained from the pulmonary artery relates to oxygen consumption and oxygen delivery in the body. Central venous oxygen saturation (ScvO_2_) obtained from central upper venous access is commonly used as a surrogate marker of SvO_2_ since research has proven that ScvO_2_ can be used in a less invasive manner to assess the balance between oxygen delivery and oxygen consumption [3].

The gold standard for ScvO_2_ measurement is the analysis of the central venous blood sample by a Co-oximeter as this is a direct measurement of the effective amount of oxygen diluted in the sample. ScvO_2_ can otherwise be inferred on standard blood gas analysis (ABG) machines by regression calculation based on the hemoglobin dissociation curve.

Previous studies have aimed at measuring the degree of agreement between these different techniques [4–6]. Most of these studies were done either in small numbers of patients, used different ABG machines for the same cohort of patients or were done in non-adult populations. They provided conflicting results regarding the use of a calculated ScvO_2_ (Calc_ScvO_2_) as a clinically acceptable surrogate of measured ScvO_2_ (Co-oximetry_ScvO_2_).

The primary aim of our study was to prospectively assess the agreement between Calc_ScvO_2_ and Co-oximetry_ScvO_2_ in an adult critically ill population in general, and in a sub-population of septic shock patients. The second aim of the study was to investigate if there is any variable that can predict a Co-oximetry_ScvO_2_ value ≥ 70% in the whole population and septic shock patients.

## Materials and methods

### Ethics statement

This prospective and observational study was conducted in a single, mixed medical, and surgical adult ICU between January and August 2017. This study was approved by the Institutional Ethics Committee (comité d’éthique du centre Hospitalier du Dr. Shaffner de Lens). As the blood tests and data collected in this study were all standard clinical practice, the requirement for informed written consent was waived, and only oral consent was obtained. There were no measures taken to document to verbal consent procedure; nevertheless, the entire consent procedure was submitted to the ethics committee before they approved this study. If the patient or his/her next of kin refused consent, the patient’s data were not entered into the analysis.

### Patients

Patients were included if they met all of the following criteria: age >18 years, and central line with the tip confirmed by x-ray to be in the superior vena cava near or at the right atrium. Exclusion criteria were pregnancy and unstable condition, the latter being defined by >10% variation in heart rate, mean arterial pressure, and the need for clinical intervention within the 30-minute period before sampling.

### Procedure

Venous blood gas samples were obtained from the central venous cannula, respectively, using a preheparinized 3-mL BG syringe (RAPIDLyte; Siemens Healthcare Diagnostic Inc, Deerfield, IL USA). As described in detail previously [7], immediately before sampling, the intravenous catheter was flushed using the standard flush solution of 0.9% sodium chloride without heparin. To reduce dilution effects, a 10-mL sample of blood was withdrawn into the syringe and discarded before drawing the 3-mL test samples. The tap in between the sampling port and administration set tubing was turned 45^0^ while changing syringes to ensure that the solution from the proximal tubing could not enter the dead-space. Air bubbles were expelled, and the syringes were cupped and analyzed immediately, with temperature correction, using the GEM Premier 4000 (Instrumentation Laboratory Co, Paris, France). Maintenance, calibration, and quality control are performed on a regular basis by the central hospital laboratory. According to the manufacturer, the coefficient of variation for the PO_2_ for the range of PcvO_2_ was 1.66 to 3.31% and the coefficient of variation for Co-oximetry_ScvO_2_ was 0.2 to 0.6%. The dead-space was 1.9 mL for the venous system.

No medical or nursing interventions were allowed while sampling was being performed.

### Data collection

Demographic data, ICU admission diagnosis, and the Simplified Acute Physiology Score were obtained on the day of enrollment. Mean arterial pressure, the ventilation type (mechanical or spontaneous), and the use of vasopressor drugs were also registered. Septic shock was defined according to the Sepsis-3 criteria [8].

Central venous oxygen tension (PcvO_2_), central venous carbon dioxide tension (PcvCO_2_), measured central venous oxygen saturation (Co-oximetry_ScvO_2_), calculated central venous oxygen saturation (Calc_ScvO_2_), central venous pH, central venous blood lactate levels, hemoglobin concentration, and central venous base excess were measured using the GEM Premier 4000 (Instrumentation Laboratory Co, Paris, France). Co-oximetry ScvO_2_ is determined by measuring the hemoglobin level of oxygen saturation based on a spectrophotometry optical system that monitors over 100 wavelengths in the absorbance spectra of oxyhemoglobin, deoxyhemoglobin, carboxyhemoglobin, and methemoglobin.

Both the Co-oximetry_ScvO_2_ and Calc_ScvO_2_ measurements were performed using the same point-of-care blood gas analyzer (GEM Premier 4000, Instrumentation Laboratory Co, Paris, France) on the same blood sample so that no additional blood withdraws was needed.

### Sample size calculation

To calculate the sample size required to investigate the agreement between Co-oximetry_ScvO_2_ and Calc_ScvO_2_ by using Bland and Altman method [9], we decided to consider that a bias of 1% between the measured and calculated ScvO_2_ with an estimated standard deviation of the difference of 1.5% [7] to be clinically pertinent and acceptable with a maximum allowed difference between the two methods of measurements of 5%. In order to achieve these requirements with a risk α of 0.05 and a power of 80%, 51 patients were needed to be included in the study.

### Statistical analysis

Data are presented as mean ±SD or as median (25–75%, interquartile range). Normality was evaluated using the Shapiro–Wilk test. Comparisons of continuous variables between septic shock and non-septic shock patients were assessed using Student’s test or Mann-Whitney test as appropriate. Comparisons of categorical variables were performed using χ^2^-test or Fisher exact test as appropriate.

Agreement between Co-oximetry_ScvO_2_ and Calc_ScvO_2_ measurements was assessed using the Bland-Altman method [10]. Other methods used to evaluate the agreement are also described. There are the intra-class correlation coefficient (ICC) [11] and the Cohen’s Kappa coefficient. According to Bland and Altman, most disagreements between measurements are expected to fall between limits called “limits of agreement” defined as d ± 1.96 SD_diff_ where d is the mean difference (bias) between the pairs of measurements, and SD_diff_ is the standard deviation of the differences [12]. The ICC equals variance between patients divided by variance between patients plus variance between measurements. The value of the ICC ranges from 0 to 1, 1 representing perfect agreement of the measurement. The Cohen’s Kappa coefficient was calculated to assess the agreement between Co-oximetry_ScvO_2_ and Calc_ScvO_2_ after categorizing the two variables into two groups using a cutoff value of 70%. The values of the ICC and Cohen’s Kappa coefficient range from 0 to 1, 1 representing perfect agreement of the measurements.

Simple linear regression analysis with the difference between Co-oximetry_ScvO_2_ and Calc_ScvO_2_ used as the dependent variable was performed, and variables with a *P*-value less than 0.2 or physiologically important were included in a multiple linear regression analysis model. Adjusted R^2^ for the final model and each variable entry along with their standardized coefficients were also provided. The final model was tested for the presence of collinearity (VIF test).

Receiver operating characteristics (ROC) curves were constructed to evaluate the ability of the most explanatory variable of the Co-oximetry_ScvO_2_ and Calc_ScvO_2_ difference (found from the multiple linear regression model) to predict a Co-oximetry_ScvO_2_ value ≥ 70% in the whole population and septic shock patients. The best cutoff of a ROC curve was chosen with the highest Youden index [12]. Sensitivity, specificity, positive and negative predictive values along with their 95% confidence intervals were calculated.

Statistical analysis was performed using SPSS for Windows release 17.0 (Chicago, Illinois, USA) and MedCalc 18.6 (MedCalc Software, Mariakerke, Belgium). *P* < 0.05 was considered statistically significant. All reported P values are two-sided.

## Results

One hundred patients were prospectively included in this study to have enough power for investigating the subgroup of septic shock patients. Basic characteristics of the cohort are presented in Table 1. The median age of patients was 66 [55–75] with a mean SAPS II score of 57±22. Forty-nine patients had septic shock, and 68% were mechanically ventilated. The comparisons of blood gas parameters between septic shock and non-septic shock patients are displayed in Table 2. Overall, septic shock patients were more acidotic and had higher lactate levels.

**Table 1:** Characteristics of the patients (n = 100).

**Age, yrs**	66 [55–75]
**Sex, (Male/Female)**	37/63
**ICU mortality, n**	37
**Mechanical ventilation, n**	68
**SAPS II**	57±22
**Septic shock, n**	49
**Norepinephrine, n**	49
**Reason for admission in ICU, n**	
**Pneumonia**	41
**Peritonitis**	30
**Pancreatitis**	8
**Cardiac failure**	7
**Status epilepticus**	6
**Others**	5
**Venous pH, (minimum, maximum)**	(7.05, 7.53)
**PcvO_2_, mmHg (minimum, maximum)**	(22, 86)
**PcvCO_2_, mmHg (minimum, maximum)**	(27, 89)
**Co-oximetry_ScvO_2_, % (minimum, maximum)**	(46, 98)
**Calc_ScvO_2_, % (minimum, maximum)**	(40, 96)
**Base Excess, mmol/L (minimum, maximum)**	(–18, +17)
**Bicarbonate, mmol/L (minimum, maximum)**	(11, 46)
**Lactate, mmol/L (minimum, maximum)**	(0.5, 13.3)
**Hemoglobin, g/dL (minimum, maximum)**	(4, 17)

ICU, intensive care unit; SAPS, simplified acute physiologic score; Co-oximetry-ScvO_2_, Co-oximetry central venous oxygen saturation; Calc_ScvO_2_, calculated central venous oxygen saturation; PcvO_2_, central venous oxygen tension; PcvCO_2_, central venous carbon dioxide tension. Data are expressed as mean ± SD, count, or median [interquartile, 25–75].

**Table 2:** Comparisons of blood gas parameters between septic shock and non-septic shock patients.

	**All patients** **(n = 100)**	**Septic shock** **(n = 49)**	**Non-Septic shock** **(n = 51)**	***P*-value**
**Mean arterial pressure, mmHg**	80±15	77±14	83±16	0.057
**Co-oximetry_ScvO_2_, %**	73±10	74±10	68±11	0.454
**Co-oximetry_ScvO_2_ < 70%, n (%)**	35 (35)	17 (35)	18 (35)	0.950
**Calc_ScvO_2_, %**	68±11	68±11	68±11	0.878
**Calc_ScvO_2_ < 70%, n (%)**	47 (47)	23 (47)	24 (47)	0.909
**(Co-oximetry_ScvO_2_ –Calc_ScvO_2_), %**	4.5±6.0	5.6±6.0	3.5±5.5	0.082
**Venous pH**	7.36 [7.32–7.42]	7.35 [7.28–7.38]	7.40 [7.33–7.43]	0.005
**PcvO_2_, mmHg**	38 [33–43]	38 [33–43]	36 [32–43]	0.269
**PcvCO_2_, mmHg**	43 [38–50]	43 [39–48]	45 [38–53]	0.159
**Bicarbonate, mmol/L**	25.4±6.2	23.0±5.8	27.7±5.7	<0.001
**Base Excess, mmol/L**	-0.3±6.3	-2.8±6.4	2.1±5.3	<0.001
**Lactate, mmol/L**	1.6 [1–2.6]	2.5 [1.5–3.9]	1.1 [0.8–1.6]	<0.001
**Hemoglobin, g/dL**	10.7±2.2	11.1±2.4	10.4±1.9	0.096

Co-oximetry_ScvO_2_, Co-oximetry central venous oxygen saturation; Calc_ScvO_2_, calculated central venous oxygen saturation; PcvO_2_, central venous oxygen tension; PcvCO_2_, central venous carbon dioxide tension. Data are expressed as mean± SD or as median [interquartile range, 25–75].

### The whole population

**Fig 1:**
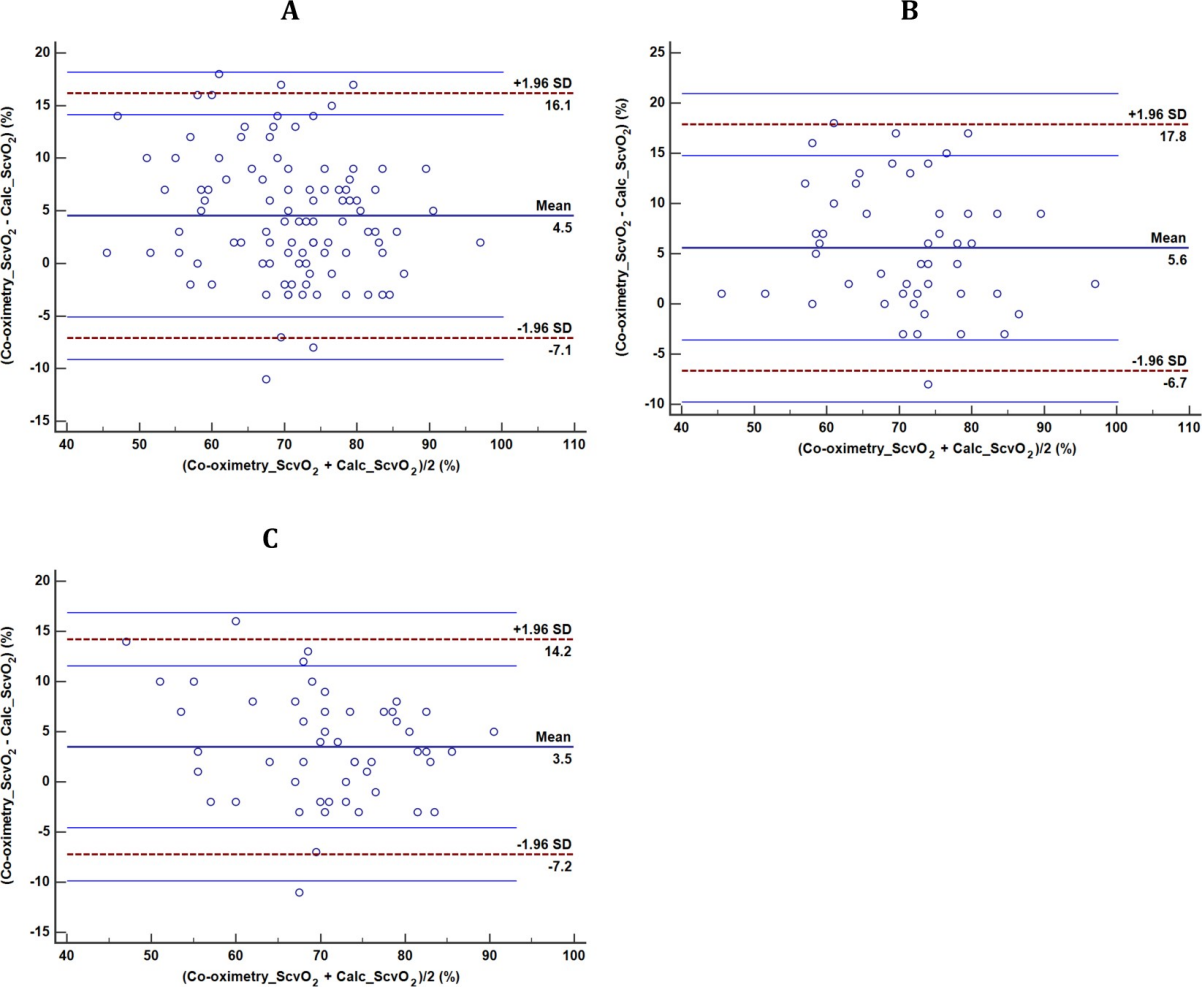
Bland and Altman plot of the difference against the mean of Co-oximetry ScvO_2_ and the calculated ScvO_2_. The solid line (dark bleu) represents the mean difference (bias) between both techniques. The dashed lines represent the upper and lower limits of agreement, whereas the solid lines (light blue) surrounding the upper and lower limits of agreement represent their 95% confidence intervals. (A) The whole population, (B) Septic shock patients, and (C) Non-septic shock patients.

Fig 1A shows the Bland-Altman diagram comparing ScvO_2_ values measured with co-oximetry and the calculated ScvO_2_ values. We found a high mean difference (bias) between the two methods (4.5±6.0%), which was significantly different from zero (P< 0.001). Furthermore, the limits of agreement were wide (-7.1, +16.1).

The agreement between the two parameters expressed as ICC was 0.754 [(95% CI: 0.393–0.880), P< 0.001]. We also calculated the agreement between the two parameters after categorized them into S_cv_O_2_ values less than 70% and higher or equal to 70%. We found a moderate agreement with a Cohen’s Kappa coefficient of 0.470 (P <0.001).

### Agreement according to septic and non-septic shock patients

In septic shock patients, the mean difference between Co-oximetry_ScvO_2_ and Calc_ScvO_2_ was 5.6±6.0% (Table 2) with limits of agreement ranged from –6.7 to 17.8% (Fig 1B). In non-septic shock patients, the mean difference between Co-oximetry_ScvO_2_ and Calc_ScvO_2_ was 3.5±5.5%, and the limits of agreement were wide from –7.2 to 14.2% (Fig 1C). There was no significant difference between the two groups regarding the mean difference between the two techniques (Table 2).

The ICC was a little bit higher in non-septic shock compared with septic shock patients (0.799 [95% CI: 0.534–0.902] vs. 0.720 [95% CI: 0.222–0.881]).

In septic shock patients, among the 23 patients with Calc_ScvO_2_ < 70%, 9 (39%) had Co-oxy_ScvO_2_ > 70% (P = 0.001). The Cohen’s Kappa coefficient was 0.501 (P <0.001). Among the 24 patients, in non-septic shock group, with Calc_ScvO_2_ < 70%, 10 (42%) had Co-oxy_ScvO_2_ > 70% (P = 0.002). The Cohen’s Kappa coefficient was 0.441 (P = 0.001).

### Factors affecting the mean difference between Co-oximetry_ScvO_2_ and Calc_ScvO_2_ in the whole population

The best multiple regression analysis model constructed from the data found that PcvO_2_, Co-oximetry_ScvO_2_, venous pH, and Hb were the independent determinants of Co-oximetry_ScvO_2_ and Calc_ScvO_2_ difference (adjusted R^2^ = 0.80; P< 0.001) (Table 3). Variables were excluded from the model if they did not change the adjusted R^2^. PcvO_2_ was the main independent explanatory variable to predict the Co-oximetry_ScvO_2_ and Calc_ScvO_2_ difference because of its highest absolute standardized coefficient and the highest changes made in adjusted R^2^ when it was entered in the model (Table 3). The model did not reveal collinearity (all VIFs were < 5 and all tolerances were > 0.2).

**Table 3:** Multiple linear regression model for the difference between co-oximetry ScvO_2_ and calculated ScvO_2_ (Co-oxy_ScvO_2_ –Calc_ScvO_2_).

**(Co-oximetry_ScvO**_**2**_ **–Calc_ScvO**_**2**_**)**	**Coefficients**	**95% CI**	***P-*value**	**Standardized Coefficients**
**Venous pH**	–65.64	–73.50 to –57.80	<0.001	–0.92
**PcvO_2_**	–1.18	–1.31 to –1.04	<0.001	–1.60
**Co-oximetry_ScvO_2_**	0.76	0.66 to 0.87	<0.001	1.19
**Hemoglobin**	–0.16	-0.42 to 0.09	0.210	–0.06

PcvO_2_, central venous oxygen tension; Co-oximetry-ScvO_2_, Co-oximetry central venous oxygen saturation; Calc_scvO_2_, calculated central venous oxygen saturation; CI, confidence interval.

### Ability of PcvO_2_ to predict Co-oximetry_ScvO_2_ ≥70%

**Fig 2:**
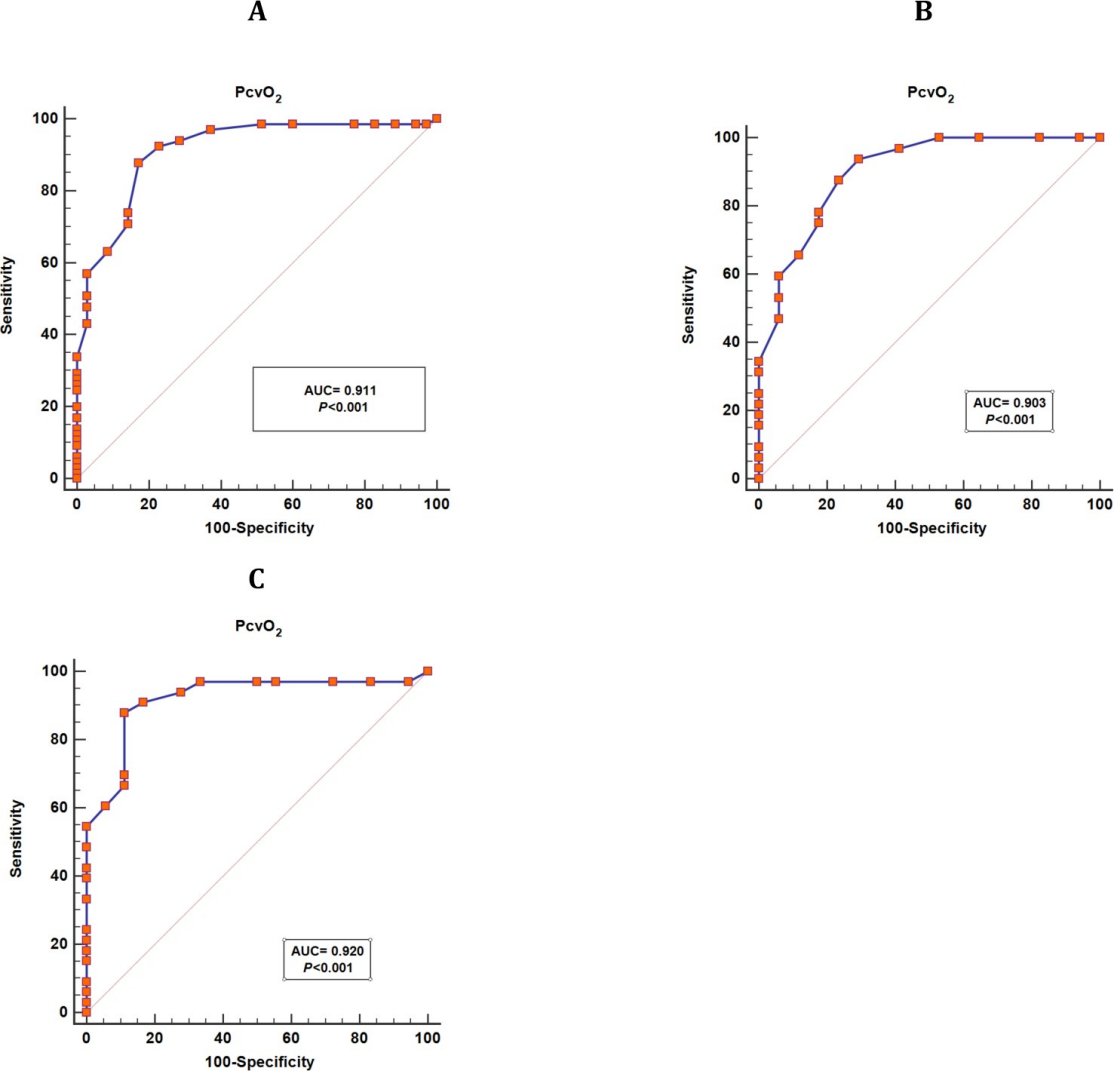
Receiver operator characteristic (ROC) curve showing the ability of the central venous oxygen pressure (PcvO_2_) to predict Co-oximetry ScvO_2_ greater than or equal to 70% in all patients. (A) The whole population, (B) Septic shock patients, and (C) Non-septic shock patients.

The ability of PcvO_2_ to predict Co-oximetry_ScvO_2_ value ≥ 70%, in all patients, was excellent with AUC of 0.911 [95% CI: 0.837–0.959] (Fig 2A). The best cutoff value was ≥36 mmHg with a sensitivity of 88% [95% CI: 77–94%], specificity of 83% [95% CI: 66–93%], positive predictive value of 90% [95% CI: 82–95%], and negative predictive value of 78% [95% CI: 65–88%] (Table 4).

**Table 4:** Predictive values of PcvO_2_ to detect Co-oximetry_ScvO_2_ value ≥ 70% in different populations. The best cutoff PcvO_2_ value was ≥36 mmHg for the whole and non-septic shock patients, and ≥ 35 mmHg for septic shock patients.

	**AUC [95%CI]**	**Se [95 CI] (%)**	**Spe [95 CI] (%)**	**PPV [95% CI] (%)**	**NPV [95% CI] (%)**
**Whole population**	0.911 [0.837–0.959]	88 [77–94]	83 [66–93]	90 [82–95]	78[65–88]
**Septic shock**	0.903 [0.784–0.969]	94[79–99]	71[44–90]	86[74–93]	86[60–96]
**Non septic shock**	0.920 [0.809–0.977]	89[72–97]	89[65–99]	94[80–98]	80[61–91]

AUC, area under the curve; Se, sensitivity; Spe, specificity; PPV, positive predictive value; NPV, negative predictive value; CI, confidence interval.

Furthermore, we found that, in septic shock patients, the ability of PcvO_2_ to predict Co-oximetry_ScvO_2_ value ≥ 70% was, also, excellent with AUC of 0.903 [95% CI: 0.784–0.969] (Fig 2B). The best cutoff value was ≥ 35 mmHg with a sensitivity of 94% [95% CI: 79–99%], specificity of 71% [95% CI: 44–90%], positive predictive value of 86% [95% CI: 74–93%], and negative predictive value of 86% [95% CI: 60–96%] (Table 4).

Also, in non-septic shock population, the ability of PcvO_2_ to predict Co-oximetry_ScvO_2_ value ≥ 70% was, also, excellent with AUC of 0.920 [95% CI: 0.809–0.977] (Fig 2C). The best cutoff value was ≥ 36 mmHg with a sensitivity of 89% [95% CI: 72–97%], specificity of 89% [95% CI: 65–99%], positive predictive value of 94% [95% CI: 80–98%], and negative predictive value of 80% [95% CI: 61–91%] (Table 4).

## Discussion

The main findings of our study were that (1) the agreement between the CO-oximetry_ScvO_2_ and the calculated ScvO_2_ was poor in all population as well as in septic shock patients; (2) PcvO_2_ was the primary independent variable that could explain the difference between ScvO_2_ measured by CO-oximetry and the calculated ScvO_2_; (3) PcvO_2_ had an excellent ability to predict CO-oximetry_ScvO_2_ values ≥ 70% in all population as well as in septic shock patients.

### Calculated and measured ScvO_2_

Venous blood gas analysis allows for the direct and accurate determination of a series of oxygen-related parameters including the partial pressure of oxygen in venous blood or PvO2. The saturation of oxygen may be directly measured by CO-oximetry, or it may be calculated.

The oxygen saturation reflects only the oxygen that is bound to hemoglobin. It is an expression of the total percentage of oxygen binding sites within the hemoglobin molecules that are occupied by oxygen. It is, in fact, a measure of the oxygen-carrying capacity that is in use. This represents almost all of the oxygen present in the venous blood (over 98%) the rest is dissolved in plasma and expressed as a partial pressure of O_2_. Even if representing just a very small percentage of the total oxygen content of venous blood the partial pressure of O_2_ is relevant as it is the main determinant of hemoglobin affinity to oxygen. This change of hemoglobin affinity is traditionally expressed by the oxygen dissociation curve where the higher the oxygen partial pressure, the higher the hemoglobin affinity becomes.

Before the current generation of blood gas analyzer machines, ScvO_2_ and arterial oxygen saturation could only be obtained by relying on the direct determination of the oxygen partial pressure and estimating the saturation using a mathematical representation of the oxygen dissociation curve. One can understand that this comes with some potential for errors as it considers that the only factor influencing the shape and position of the dissociation curve is PvO_2_. However, other factors, namely (5): temperature, pH, the partial pressure of CO_2_, the concentration of non-oxygen binding hemoglobin (carboxyhemoglobin and methemoglobin) and the concentration of 2,3-diphosphoglycerate also affect the oxygen dissociation curve. Furthermore, the influence of 2,3-diphosphoglycerate, a three-carbon isomer of the glycolytic intermediate 1,3-bisphosphoglyceric acid, maybe even more relevant at lower oxygen partial pressures since it binds with higher affinity to deoxygenated hemoglobin [13,14].

The use of the mathematical equivalent of a standard hemoglobin dissociation curve also assumes standard conditions namely a pH of 7,4, temperature of 37°C, partial pressure of CO_2_ of 40 mmHg, normal concentrations of 2,3-diphosphoglycerate and normal levels of methemoglobin and carboxyhemoglobin. Some changes in these parameters such as higher temperature, higher CO_2_ partial pressure, acidosis, increased 2,3-diphosphoglycerate, will displace the curve to the right (lower saturation at a given PcvO_2_). The opposite changes of these parameters: lower temperature, lower CO_2_ partial pressure, alkalosis, low 2,3-diphosphoglycerate and higher concentrations of others hemoglobin will have the opposite effects by displacing the curve to the left, which means higher saturation for a given partial pressure).

The blood gas analyzer machine will attempt to mitigate these sources of errors by employing complex algorithms and taking into account more than just PcvO_2_. It requires the input of pH, PcvCO_2_, temperature and calculated base excess. No model integrates the concentration of 2,3-diphosphoglycerate or concentrations of the others hemoglobin to calculate ScvO_2_. The inaccuracy resulting from these models was shown in arterial samples by Gothgen et al. almost thirty years ago [15]. There might an even more significant potential for errors in the critical illness where patients have severe acid-base or temperature disturbances and are possibly hypoxemic and also when using venous samples that have lower PO_2_ values than their arterial counterparts.

Modern blood gas analyzers also allow for the direct measurement of oxygen saturation: CO-oximetry is based on spectrophotometric analysis of blood. Spectrophotometry is a tool that hinges on the quantitative analysis of molecules depending on how much light is absorbed by colored compounds. It was first applied for dosing of total hemoglobin concentration in blood [16]. The multiple subspecies of hemoglobin present in blood, oxyhemoglobin, deoxyhemoglobin, methemoglobin, and carboxyhemoglobin have each a specific light absorption and transmission wavelength and can thus be quantified [17].

Knowing the measured oxygenated and non-oxygenated hemoglobin concentrations, one can directly deduce the saturation of oxygen in the sample as follows: SvO_2_ = cO_2_Hb/(cO_2_Hb + HHb) where cO_2_Hb is the oxyhemoglobin concentration in venous blood and cHHb is the concentration of deoxyhemoglobin in venous blood.

Contrary to the calculated oxygen saturation, the measured by CO-oximetry is not dependent on pH, temperature, hemoglobin concentration, 2,3-diphosphoglicerate or any other parameter that may displace the hemoglobin dissociation curve.

### Agreement between CO-oximetry_ScvO_2_ and Calc_ScvO_2_

We found significant differences between measured and calculated ScvO_2_ for this study population. The Bland and Altman plot analysis shows a large bias and wide limits of agreement (Fig 1A). Also, the interclass correlation coefficient (ICC) was found to have a large confidence interval, which is in favor of non-agreement between CO-oximetry_ScvO_2_ and Calc_ScvO_2_. Moreover, the Cohen’s Kappa coefficient, which measures the inter-rater agreement for qualitative (categorical) items, and is believed to be a more robust test than the simple percent agreement calculation was low. This, also, points for lack of agreement between the two methodologies.

One might suppose that this disagreement in methods would be limited to extreme physiological conditions as in shock. We found that, indeed, the septic shock sub-group was significantly different in various physiological variables that impact the hemoglobin dissociation curve, namely pH, venous bicarbonate concentration and venous lactate concentration. Our results show poor agreement between Co-oximetry_ScvO_2_ and Calc_ScvO_2_ in septic shock patients (Fig 1B). Overall, we have demonstrated that the discrepancy between the measured and calculated ScvO_2_ extends to all patient groups regardless of shock (Fig 1C).

Our findings are in line with previous results [5,6]. Indeed, a prior study by Romero et al. [5] in 16 septic shock patients with 111 pairs of measurements also showed lack of agreement between Co-oximetry_ScvO_2_ and calculated ScvO_2_ with wide apart limits of agreement on Bland and Altman plot analysis. A recent study using 141 paired samples from 82 pediatric ICU patients by Subramanian et al. [6] also failed to show adequate agreement between the two methodologies. Inversely, only one earlier study [4], which included 28 critically ill patients with 46 pairs measurements, found an insignificant systematic difference between measured and calculated ScvO_2_ (0.78%) with smaller limits of agreement (-5.52 to 4.96%). However, the characteristics of the population were not provided in that study [4].

We should also consider that this discrepancy between measured and calculated ScvO_2_ could have immediate clinical and treatment consequences. Indeed, 23 patients in septic shock would have presumably needed new interventions (possibly fluids or additional vasopressor) because their Calc_ScvO_2_ values were < 70% when these same patients were found to be within the recommendations targets of a measured ScvO_2_ over 70%. Likewise, for the non-septic shock population, 24 patients would be erroneously classified as having a low ScvO_2_ (Co-oximetry_ScvO_2_).

Four independent variables, in a multiple linear regression model, were found to be the main determinant of the disagreement between CO-oximetry_ScvO_2_ and Calc_S_cv_O_2_ with PcvO_2_ being the most explanatory variable with the highest standardized coefficient (Table 4). Our multiple regression’ model proved to be strong as it had a high adjusted R^2^ (0.8). With an AUROC of 0.911, PcvO_2_ found to be an excellent predictor for CO-oximetry_ScvO_2_ value above 70%. A cut-off ≥ 36 mmHg was demonstrated to be the best discriminative value for all population and ≥ 35 mmHg for septic patients. Our results are also in agreement with previously published data by Romero et al. [5] who found, in septic shock patients, an excellent AUROC (0.87) but a little different cut-off point (40 mmHg) to predict CO-oximetry_ScvO_2_ values > 70%. From a practical standpoint, considering that CO-oximetry_ScvO_2_ is not readily available in all institutions and that the primary intent of a physician when ordering a test aimed at determining venous oxygen saturation is to know if the patients saturation is under or over the 70% threshold, we found that using PcvO_2_ is highly specific and sensitive to predict CO-oximetry_ScvO_2_ above 70%.

To our knowledge, this is the first prospective study comparing calculated saturation and CO-oximetry in a large ICU population of septic shock and non-septic shock patients. The strengths of our study compared to the others [4–6] are: (1) only 1 measurement per patient was performed whereas the other studies [4,5] included fewer patients with multiple measurements per patient without adjusting for that [18], introducing potential errors in the results; (2) Co-oximetry_ScvO_2_ and Calc_ScvO_2_ values were provided by the same point-of-care blood gas analyzer (GEM 4000) while in the other studies two different machines were used to compare the two methodologies, which could increase the pre-analytical errors (by increasing the waiting time for each sample to be analyzed by two devices) and the analytical errors related to each machine; (3) we used different methods to examine the agreement between the two variables, and sample size calculation with power analysis was performed.

Our findings are of clinical importance. Indeed, our results are a step forward in raising awareness that these two methods of determining ScvO_2_ are not equivalent in any circumstance in the ICU population and following calculated saturation may lead to diagnosis missteps and unwarranted therapeutic interventions in almost half of our patients.

Some limitations must be recognized for our study. First, it took place in a single center using a unique brand and model of analyzer. Second, we did not process 2,3-diphosphoglycerate concentrations and temperature levels. However, the venous blood gas variables were corrected for temperature level, and no one of our patients was profoundly hypo or hyperthermic. Also, our multiple linear regression model was very good (adjusted R^2^ = 0.8) without including temperature and 2,3-diphosphoglycerate levels. Furthermore, our findings are in line with the results of other studies [5,6].

## Conclusion

No agreement was found between the measured and calculated ScvO_2_ in the whole population as well as in septic shock patients. Our results do not recommend the use of calculated ScvO_2_ to guide resuscitation in critically ill patients. In situations where the Co-oximetry technique is not available, relying on PcvO_2_, measured by any blood gas analyzer, to predict the Co-oximetry_ScvO_2_ value above or below 70% could be an option.

## Supporting information

S1 File Datasets supporting the conclusions of this article. (XLSX)

## References

[1] Rhodes A, Evans LE, Alhazzani W, Levy MM, Antonelli M, Ferrer R, et al Surviving Sepsis Campaign: International Guidelines for Management of Sepsis and Septic Shock: 2016. Intensive Care Med. 2017; 43(3): 304–377. 10.1007/s00134-017-4683-6 28101605

[2] Magilligan DJ Jr, Teasdall R, Eisinminger R, Peterson E. Mixed venous oxygen as a predictor of cardiac output in the postoperative cardiac surgical patient. Ann Thorac Surg 1987; 44(3): 260–262. 3632111 10.1016/s0003-4975(10)62068-1

[3] Dueck MH, Klimek M, Appenrodt S, Weigand C, Boerner U. Trends but not individual values of central venous oxygen saturation agree with mixed venous oxygen saturation during varying hemodynamic conditions. Anesthesiology 2005; 103(2): 249–257. 16052106 10.1097/00000542-200508000-00007

[4] Johnson PA, Bihari DJ, Raper RF, Haughton MA, Fisher MM, Herkes RG. A comparison between direct and calculated oxygen saturation in intensive care. Anaesth Intensive Care. 1993; 21(1): 72–5. 8447611 10.1177/0310057X9302100117

[5] Romero CM, Luengo C, Tobar E, Fábrega L, Vial MJ, Cornejo R et al Central venous saturation in septic shock: co-oximetry vs gasometry. Am J Emerg Med. 2014; 32(10): 1275–7. 10.1016/j.ajem.2014.07.027 25171795

[6] Subramanian G, Anitha VP, Ranjit S. Comparison of central venous saturation by standard ABG machine versus co-oximeter: Is 18 carat as good as the 24 carat gold standard? Indian J Crit Care Med. 2013; 17(2): 82–6. 10.4103/0972-5229.114824 23983412 PMC3752872

[7] Mallat J, Lazkani A, Lemyze M, Pepy F, Meddour M, Gasan G et al Repeatability of blood gas parameters, PCO2 gap, and PCO2 gap to arterial-to-venous oxygen content difference in critically ill adult patients. Medicine (Baltimore). 2015; 94(3): e415 10.1097/MD.0000000000000415 25621691 PMC4602629

[8] Singer M, Deutschman CS, Seymour CW, Shankar-Hari M, Annane D, Bauer M et al The Third International Consensus Definitions for Sepsis and Septic Shock (Sepsis-3). JAMA. 2016; 315(8): 801–10. 10.1001/jama.2016.0287 26903338 PMC4968574

[9] Lu MJ, Zhong WH, Liu YX, Miao HZ, Li YC, Ji MH. Sample size for Assessing Agreement between Two Methods of Measurement by Bland-Altman Method. Int J Biostat. 2016; 12(2). 10.1515/ijb-2015-0039 27838682

[10] Bland JM, Altman DG. Statistical methods for assessing agreement between two methods of clinical measurement. Lancet 1986; 1 (8476): 307–10. 2868172

[11] Shrout PE, Fleiss JL. Intraclass correlations: uses in assessing rater reliability. Psychol Bull. 1979; 86(2): 420–8. 18839484 10.1037//0033-2909.86.2.420

[12] Ray P, Le Manach Y, Riou B, Houle TT. Statistical evaluation of a biomarker. Anesthesiology 2010; 112(4): 1023–40. 10.1097/ALN.0b013e3181d47604 20234303

[13] Bellingham AJ, Detter JC, Lenfant C. Regulatory mechanisms of hemoglobin oxygen affinity in acidosis and alkalosis. J Clin Invest. 1971; 50(3): 700–6. 10.1172/JCI106540 5545127 PMC291978

[14] Riggs TE, Shafer AW, Guenter CA. Acute changes in oxyhemoglobin affinity. Effects on oxygen transport and utilization. J Clin Invest. 1973; 52(10): 2660–3. 10.1172/JCI107459 4199605 PMC302527

[15] Gøthgen IH, Siggaard-Andersen O, Kokholm G. Variations in the hemoglobin-oxygen dissociation curve in 10079 arterial blood samples. Scand J Clin Lab Invest Suppl. 1990;(203):87–90. 2089624 10.3109/00365519009087495

[16] Davis GE, Sheard C. The spectrophotometric determination of hemoglobin. Arch Intern Med (Chic). 1927; 40(2): 226–236.

[17] Horecker BL. The absorption spectra of hemoglobin and its derivatives in the visible and near infra-red regions. J. Biol. Chem. 1943 148: 173–183.

[18] Bland JM, Altman DG. Agreement between methods of measurement with multiple observations per individual. J Biopharm Stat. 2007; 17(4): 571–82. 10.1080/10543400701329422 17613642

